# ADVANCING PROGRAM REACH BY TAKING TRAINING ONLINE

**DOI:** 10.1093/geroni/igz038.2465

**Published:** 2019-11-08

**Authors:** Andrew DeMott, Susan Hughes, Lesha Spencer-Brown

**Affiliations:** 1 University of Illinois at Chicago, Chicago, Illinois, United States; 2 National Recreation and Parks Association, Ashburn, Virginia, United States

## Abstract

The National Recreation and Park Association (NRPA) is one of four national organizations to receive funding from CDC to disseminate arthritis-appropriate evidence-based interventions (AAEBIs). NRPA has partnered with Fit & Strong!, a CDC-approved AAEBI and National Council on Aging falls prevention program, to disseminate through local park and recreation agencies across the country. To meet the needs of this dissemination effort, Fit & Strong! adapted its in-person instructor training into an online curriculum which became available March 2018. After initial pilot-testing, Fit & Strong! created a five-module training curriculum using Moodle teaching software. The training curriculum uses slides, graphics, videos, and interactive questions to assess mastery. After completing the training, fidelity checks are performed on all instructors by having them video record a class session and share it with the Fit & Strong! team for review. After evaluating the videos, the team meets with the instructors by phone to debrief and advise on any needed improvements. Through this partnership, Fit & Strong! trained an initial cohort of 39 instructors across 18 states within a month’s time. Training evaluation results have been positive. To date, over 900 participants have been reached. A second cohort of approximately 40 new instructors will be trained in Spring 2019. This presentation will review the processes of creating and executing the online training, potential cost and time-saving benefits to CBOs, instructor feedback, and the ease with which instructors can complete the training and offer the program anywhere across the country.

